# Bridge Crack Inspection Efficiency of an Unmanned Aerial Vehicle System with a Laser Ranging Module

**DOI:** 10.3390/s22124469

**Published:** 2022-06-13

**Authors:** Szu-Pyng Kao, Feng-Liang Wang, Jhih-Sian Lin, Jichiang Tsai, Yi-De Chu, Pen-Shan Hung

**Affiliations:** 1 Department of Civil Engineering, National Chung Hsing University, Taichung 40227, Taiwan; spkao@dragon.nchu.edu.tw (S.-P.K.); a13627g@gmail.com (J.-S.L.); 2 Department of Electrical Engineering, National Chung Hsing University, Taichung 40227, Taiwan; jichiangt@nchu.edu.tw (J.T.); i0938680393@gmail.com (Y.-D.C.); 3 Department of Land Management, Feng Chia University, Taichung 407301, Taiwan; pshung@fcu.edu.tw

**Keywords:** unmanned aerial vehicle, bridge inspections, crack identification, digital image processing

## Abstract

In this study, an unmanned aerial vehicle (UAV) with a camera and laser ranging module was developed to inspect bridge cracks. Four laser ranging units were installed adjacent to the camera to measure the distance from the camera to the object to calculate the object’s projection plane and overcome the limitation of vertical photography. The image processing method was adopted to extract crack information and calculate crack sizes. The developed UAV was used in outdoor bridge crack inspection tests; for images taken at a distance of 2.5 m, we measured the crack length, and the error between the result and the real length was less than 0.8%. The developed UAV has a dual-lens design, where one lens is used for bridge inspections and the other lens is used for flight control. The camera of the developed UAV can be rotated from the horizontal level to the zenith according to user requirements; thus, this UAV achieves high safety and efficiency in bridge inspections.

## 1. Introduction

**Figure 1:**
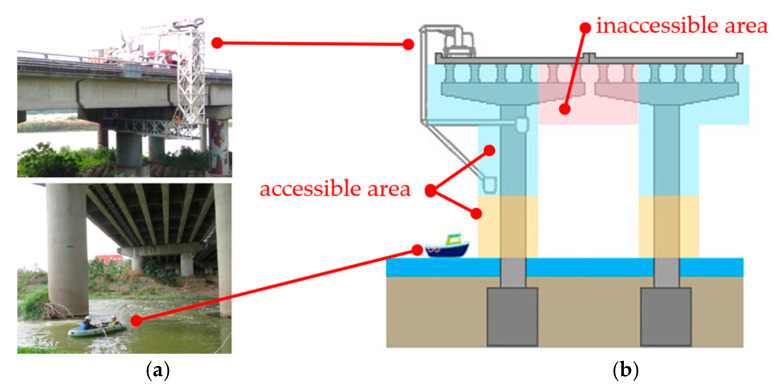
Traditional bridge inspection operations. (**a**) Bridge inspection equipment (**b**) Bridge inspection area.

Countries worldwide mostly use visual inspection methods for bridge structure inspection. For bridges with high piers and those that run across rivers and are difficult to approach, professionals must use equipment such as large bridge inspection vehicles with lifting platforms, boats, and ladders to perform detailed inspections. Such inspections are technically complex and difficult and increase the job risks for professionals [1]. Moreover, for bridges that are considerably tall or wide, bridge inspection vehicles are unsuitable for structural inspections. In many situations, such as those in Figure 1a,b, during traditional bridge inspection operations, the inspectors need to use a ruler to measure the length and the width of the bridge crack. Under this condition, the maximal value is 1 m. If the distance between the inspector and inspected surface is larger than this, the inspectors cannot measure the bridge crack; they can only take an image of the crack. If the distance is larger than 6 m, it is hard to take a clear image and recognize it with human eyes. This influences the completeness and correctness of the data from bridge inspections. (Figure 1). Although visual inspections are essential for bridge-planning-related tasks that ensure bridge availability and safety (e.g., assessing the current deterioration conditions of bridges and performing bridge maintenance), these inspections have many disadvantages [2]. Thus, following the development of unmanned aerial vehicles (UAVs), researchers and professionals have investigated the feasibility of combining UAVs with digital photo processing technology to overcome the problems involved in visually inspecting concrete cracks [3,4,5]. UAVs can capture images of the surface cracks in civil engineering structures from a closer distance, which enables relevant personnel to more accurately identify cracks [6]. Bridge inspection through UAVs outperforms traditional bridge inspection methods in two aspects. First, areas that are difficult to examine using traditional bridge inspection methods can be examined using UAVs. Second, images can be collected more cheaply, safely, and flexibly when using UAVs than when using traditional bridge inspection methods [7]. Chan et al. [8] indicated that UAVs have considerable potential in performing bridge condition inspections, and UAVs ensure that inspectors do not need to be within a close range from the bridge when conducting a visual assessment.

The number of images captured by UAVs during bridge inspections is markedly large. Thus, to effectively extract crack information from images, scholars have proposed using various image-processing techniques. These techniques can roughly be divided into four categories: grayscale thresholding algorithms [9], edge-detection algorithms [10], graph-theory-based algorithms [11,12], and machine-learning algorithms [13,14,15,16,17,18]. Although these methods have advantages in identifying cracks, the size of cracks in images is given in the unit of pixels, without any information about the image-taking distance. Therefore, it is impossible to calculate the metric size of the cracks (length and width in the metric unit; crack length means the longest distance along the crack from one end to the other, and the crack widths mean the maximum vertical distance from the crack edge). Consequently, some scholars have attempted to use range-finding equipment to directly obtain photo-shooting distances. For example, Zhong et al. [19] employed an octocopter UAV that used a laser range finder installed on its camera to measure object distances and pixel resolutions. There are two main problems in the bridge inspection task: collecting data with the required information (for example, distance to object) and crack recognition in images. Well-performing data collection and image processing methods are necessary to obtain satisfactory bridge inspection results. Tian et al. [20] studied a data collection system equipped with a camera and a laser range finder and used the system to conduct laboratory tests on concrete beams with cracks. A laser range finder (range precision = ±2 mm) with the laser beams aligned parallel to the camera and perpendicularly to the object was used to determine the accurate distance between the camera and the object. The captured images were calibrated and used to produce grayscale images, which were then subjected to thresholding conducted using the Otsu method [21]. Subsequently, a combination of Canny operators was employed for edge detection [22], which allowed for the accurate identification of crack edges. Finally, the object distance method, whose calculation is based on optical triangular similarity theory and involves converting pixel size into metric system units, was used to calculate crack size in metric system units. The results verified the system’s accuracy in measuring cracks with a width larger than 0.1 mm and revealed a 92% accuracy in crack length measurement. Kim et al. [23] adopted UAVs integrated with cameras and sound-wave range finders and studied tests performed on-site on the concrete wall of a sports center. An ultrasonic displacement sensor (range precision = ±3 mm) was used for the tests; the sensor was aligned in parallel to the camera and perpendicularly to the wall to accurately measure the distance between the camera and the object. The captured images were calibrated and used to produce grayscale images. These images were then subjected to Sauvola local thresholding, which involved binarizing the images with two parameter combinations that had the lowest estimation error for the actual crack length and width. Next, skeletonization and edge detection were performed to extract the skeleton and contour pixels. The length and width were determined by measuring the length of the main crack axis and the width of the nearest contour pixels, respectively, on the two sides of the main crack axis. Finally, the object distance method was used to convert the pixel size into metric system units to calculate the crack dimensions in metric system units. According to the result, the measurement method provided accurate measurements for cracks with a width larger than 0.25 mm and had a 96.7% accuracy in measuring crack length. However, in this approach, only one range sensor is used to obtain the photo-shooting distance. Thus, assuming that the cameras are perpendicular to the targets, measurement errors occur if cracks are tilted, which makes the approach impractical.

To overcome the limitations of vertical photo-shooting, in this study, a UAV system that contains a camera and laser ranging module was designed and developed to inspect the side and bottom areas of bridge structures. These areas are dimly lit narrow spaces between bridge beams and columns, from which satellite positioning signals cannot be obtained. A tripod head was installed on the developed UAV, and a camera was mounted on the tripod head. Four laser ranging modules were installed adjacent to the camera (called the “inspection camera” in this study) to measure the distance from the camera to the object to calculate the projection plane of the object and accurately calculate the position of the inspection camera relative to the bridge and the metric image scales. Moreover, the image-processing method (the image-processing methods that we used are the Grayscale threshold algorithm and the graph theory, including the Sauvola local thresholding method, Zhang–Suen Skeletonization algorithm, and Erosion in Morphology, etc.) was used to extract crack information and calculate crack sizes, which ensured the integrity of the tracking and safety assessments for bridge cracks. The contributions of this paper are as follows:

(1) This study developed an adapted UAV for bridge inspection operations and the inspection camera. The camera is installed on the tripod head and can rotate from 90° (horizontal plane) to 180° (zenith), which enables the UAV to inspect the sides and bottom of bridges.
(2) This study developed the architecture and method used to integrate the camera and the laser ranging module on the embedded system (Raspberry Pi 4). Additionally, the object projection measurement method was proposed, which can overcome the limitations of vertical photography.
(3) This study proposed an image-processing method and process to extract crack information and metric size.

The rest of this article is structured as follows: In Section 2, we indicate the development and research methods used for the system, explaining the design and development of the hardware structure of the bridge inspection UAV system. We also describe the methods used to integrate the camera and laser ranging modules and the measuring accuracy test. Furthermore, we show the methods used for crack recognition and measurements. Next, in Section 3, we conduct a performance and accuracy analysis for the bridge inspection operation for the UAV system that integrates the camera and laser ranging modules. Finally, in Section 4, we conclude with the contributions of this study and its future developments.

## 2. Materials and Methods

### 2.1. Design and Development of a UAV System for Bridge Inspection

As bridges are located in diverse environments and multi-copter UAVs are prone to environmental interference, UAVs must be miniaturized to allow for them to reach as close as possible to the bridge structures and to reduce spatial constraints and wind pressure during bridge inspections. Furthermore, a UAV’s motor speeds must be sufficiently high to enhance its agility and wind resistance. Lighting equipment must be installed on UAVs to enable them to clearly view structural conditions.

**Figure 2:**
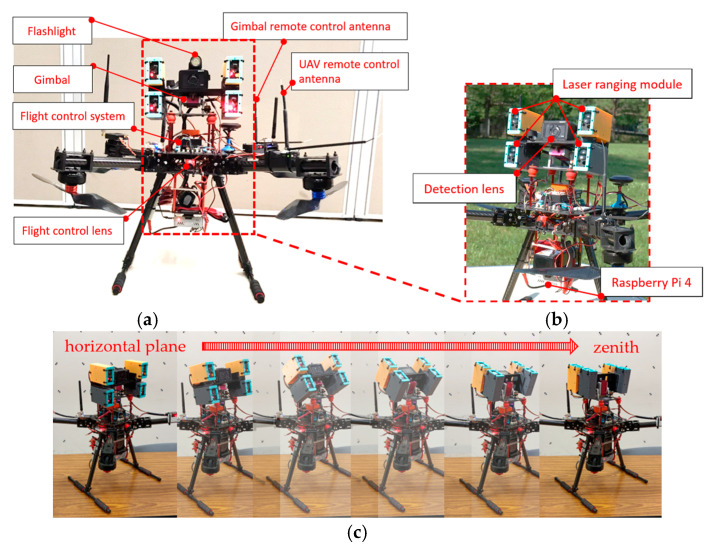
Self-assembled unmanned aerial vehicle (UAV) system for bridge inspection: (**a**) architectural diagram of the UAV; (**b**) architectural diagram of the inspection lens; (**c**) inspection camera from horizontal plane rotated to zenith.

In this study, a light UAV with a wheelbase of 63 cm was designed to clearly photograph the deteriorations in various bridge components and make relevant measurements close to the components. To prevent the UAV’s propeller from colliding with the main beams at the bottom of bridges and the subsequent crashing of the UAV, the propeller was installed under the main shaft of the UAV, which improves the UAV’s safety and convenience of operation under bridges. The developed UAV comprises three basic components: a camera (SONY DSC-RX0), a ranging module (Lidar-100), and an embedded system (i.e., Raspberry Pi 4; Figure 2a). The tripod head installed on the body of the UAV can rotate from 90° (horizontal plane) to 180° (zenith), which enables the UAV to inspect the sides and bottom of bridges. The camera of the UAV has a dual-lens design, where one lens is used for inspections and the other lens is used for UAV control. The inspection lens is placed on the tripod head (Figure 2b,c), whereas the control lens is placed in front of the UAV to enable the UAV to inform users about its dynamic conditions, thereby ensuring flight safety. Pixhawk2 Cube Orange is used as the data transmission and flight control system. It offers a one-button return for launch and route-planning functions, and considerably enhances the operation efficiency of aerial photography. The maximal effect distance at which the UAV digital remote-control equipment can transmit a video can reach 3 km, which makes it suitable for inspecting large or cross-river bridges.

**Figure 3:**
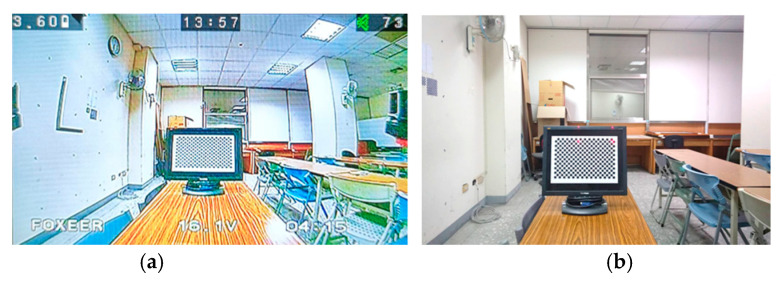
Camera’s field of view and lens distortion. (**a**) original UAV camera image (**b**) inspection camera image.

When we process and calculate the images taken by this camera, low levels of lens distortion, a long focus and high image resolution are the main factors. The UAV camera, which is market-sell or self-assembled, is usually used with a fish-eye lens. This allows us to obtain a wider field of view and more conveniently control the UAV. This could lead to more lens distortion, causing serious deformations in the image (Figure 3a). We chose the inspection camera without a fish-eye lens. The lens distortion is smaller (Figure 3b), and image deformation is low. The lens distortion can be improved by some camera methods, but the effect will decline according to the severity level of the lens distortion. In addition, in order to quickly transport real-time images to the ground station, the image resolution obtained with an original UAV camera is small. Taking our system as an example, there are specifications for original and inspection cameras (Table 1). With a photo-shooting distance of 2 m, the pixel size of projections on the object plane is 6.192 mm with an original UAV camera, but 0.582 mm with an inspection camera. This means that the spatial resolution accuracy is improved by about 10%.

With increases in the UAV the weight of the UAV, the UAV’s flight time shortens. This is a big challenge in UAV development. We used the lithium polymer (LI-PO) battery. Its capacity is 5200 mah, weight is 406.8 g, and flying time is 30 min. After adding the camera and laser ranging module to the UAV, the flight time is only 16 min. We can still add about 200 g to achieve the maximum limit. Therefore, we used the lithium iron phosphate (LFP) battery. Its capacity is 9000 mah and its weight is 577.8 g. The weight increases by 171 g, but the flight time is 23 min. This can increase by 7 min, with an improvement of about 10%. Under the condition that the UAVs are the same size, the battery is the key factor to increase the UAV’s flight time. The battery develops each day. In the future, we will obtain a battery with a lower weight and higher capacity. This will support a long flight and improve the efficiency of UAV applications.

### 2.2. Integration of the Camera and a Laser Ranging Module

**Figure 4:**
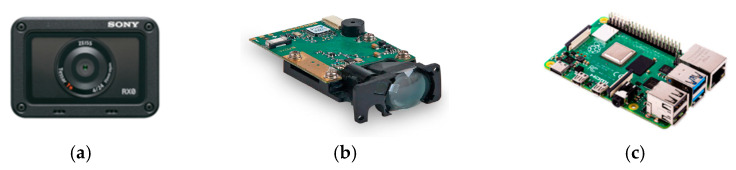
Hardware used in the integrated design [24,25,26]. (**a**) Camera (**b**)Laser ranging module (**c**) Raspberry Pi 4.

**Figure 5:**
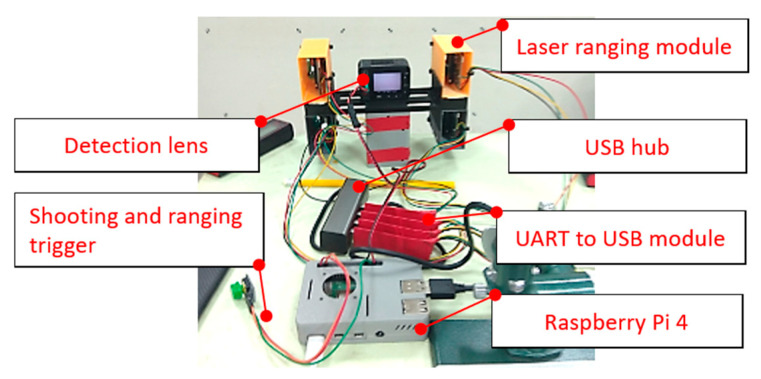
Architecture of the camera and laser ranging modules.

As shown in Figure 4a–c, small laser ranging modules (with a self-designed outer casing manufactured through three-dimensional (3D) printing) are installed at the upper left, lower left, upper right, and lower right corners of the camera to determine the relative position and attitude of the camera when capturing bridge photographs. Raspberry Pi 4 is used to collect and control camera and laser ranging modules. For integration, we used some mechanical constructions, as displayed in Figure 5.

#### 2.2.1. Synchronization Mechanism for the Operation Time of the Camera and Laser Ranging Modules

When using UAV photos to measure crack information, it is crucial to ensure that the photo-shooting and ranging processes are temporally synchronized. To synchronize the operation times of the camera and laser ranging modules, Raspberry Pi 4 was used as the control system, and buses such as GPIO, UART, I2C, and SPI were used as the Raspberry Pi 4 pinouts. The system employed in this study mainly used a GPIO as its pinout to receive the data transmitted by the laser ranging modules through a UART. The laser ranging modules transmit data to a USB module through the UART, which transmits the data to Raspberry Pi 4 by connecting four transfer modules to the USB hub.

When the USB hub is connected to Raspberry Pi 4, under the Raspberry Pi 4 Linux operating system, the Synchronous operation mode, which is programmed in Python and used to control the camera and laser ranging modules, will create four systematic abstract files (i.e., ttyUSB0, ttyUSB1, ttyUSB2, and ttyUSB3) stored in the dev folder of the root directory. The four files each represent the four connected rangefinders, and the baud rate of UART, which is used to communicate with the rangefinders and Raspberry Pi 4, is 19200 bits per second. Raspberry Pi 4 controls the camera by using the camera release cable as the command transmission cable to take pictures. The downstream end of the camera release cable uses the micro-USB, which includes a shutter (red wire), a focus (white wire), and two ground (yellow wires). First, the yellow wire was connected to the ground of the Raspberry Pi 4. Next, the white and red wires were connected to the GPIO pin of the Raspberry Pi 4. Then, a low potential was output through the GPIO of the Raspberry Pi 4 to control the camera and take pictures.

**Figure 6:**
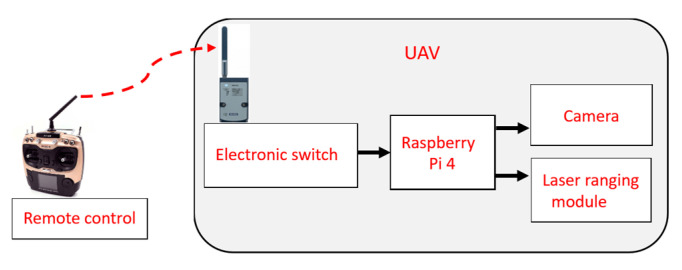
System control signal procedure.

The operation process of the system is as follows: Raspberry Pi 4 automatically executes the system program when it is turned on. Subsequently, the rangefinders and camera are turned on. The UAV remote control is used to connect to the electronic switches through the radio to drive Raspberry Pi 4, then simultaneously captures photos and makes ranging measurements. Once the UAV remote control transmits a command, Raspberry Pi 4 will execute an action, so that the writing frequency can be adjusted according to requirements during bridge inspection. When the image is received, the ranging value and time are recorded in the text file (*.txt) to calculate the projection plane (Figure 6).

#### 2.2.2. Camera Calibrations

In this study, a general commercial digital camera was used, rather than a metric camera. Therefore, the camera was not calibrated. Compared with metric cameras, digital cameras exhibit more serious system errors, such as higher lens distortion and the image plane not being perpendicular to the main shaft. Thus, the adopted camera had to be calibrated to enable it to make accurate measurements. After the camera was calibrated, correction parameters were used to calculate the system error at each “point” and added to each set of original image coordinates to satisfy measurement-accuracy-related requirements. The camera was calibrated using MATLAB Calibrator Toolbox to correct the camera lens distortion and problems related to the principal point shift, and the upper left of an image was defined as the original image coordinates. The unit of measurement was pixels. By contrast, if photogrammetry was used, the unit of measurement was given in millimeters. This means that a resection would have to be conducted for image positioning to convert the image coordinates, and the principal point would be set as the image origin. Calibrator Toolbox is easy to operate and can calibrate the values of inner orientation parameters. When using the Calibrator Toolbox, users must follow photography principles such as using specific calibration boards, placing the camera and the surface shot at an angle between 0° and 45° in relation to each other, placing the calibration board on a plane, ensuring that calibration boards are uniformly distributed in the images, and keeping the cameras in fixed focus. These steps allow for the values of the inner orientation parameters (e.g., principle point and focal length) and lens distortion difference parameters (e.g., the radiation distortion difference parameters *k*1 and *k*2, as well as the centrifugal distortion difference parameters *p*1 and *p*2) to be obtained after calibrations [27]. The camera elements of interior orientation and lens distortion calculation results are shown in Table 2.

#### 2.2.3. Overall Structural Calibrations for the Camera and Laser Ranging Modules

**Figure 7:**
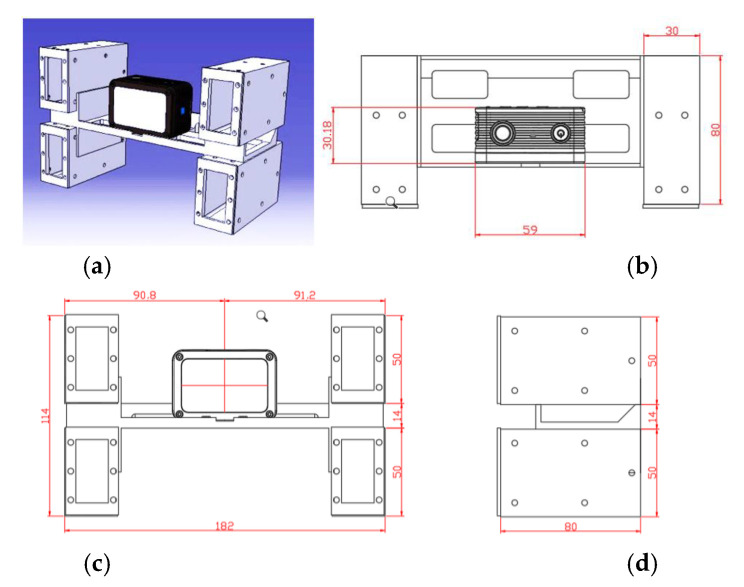
Structural dimensions of the camera and laser ranging modules: (**a**) three-dimensional image; (**b**) top view; (**c**) front view; (**d**) left view.

The integrated structure was designed after considering the shapes of the camera and laser ranging modules and ensuring that they could easily be installed on the UAV tripod head. The structure was manufactured through 3D printing (Figure 7).

**Figure 8:**
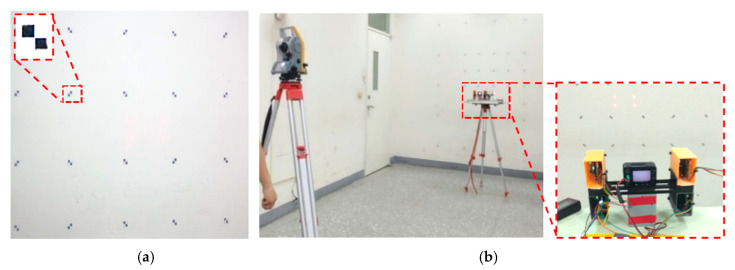
Calibrations for the control points, camera, and laser ranging modules. (**a**) Control points (**b**) Measurements made using the total station.

The spatial relationships between the laser beam vectors of the ranging modules and the camera focus have crucial effects on the measurement methods for the developed system. Therefore, a calibration procedure was designed to investigate the aforementioned spatial relationships. The steps in this procedure were as follows:

(1) The positions of the ranging modules were adjusted so that the four laser beams were nearly parallel to each other. Subsequently, the measurement accuracy was increased by measuring the laser beam vectors after calibration.
(2) As the spatial relationships between the laser beam vectors and the camera focus could not be directly measured, control points were marked on the wall of a research room, and relative spatial coordinates were measured using a total station. The room served as the system calibration site. Photos containing laser light spots and control points were captured from different distances (Figure 8a), and photogrammetry was used to calculate the spatial relationships between the laser beam vectors and the laser light spots, as well as those between the laser beam vectors and the control points. The experimental distance was increased from 1 to 3 m in 0.5-m increments. To demonstrate that the developed system can capture images with different attitudes, images were captured in five postures (i.e., facing forward, tilted to the left, tilted to the right, tilted up, and tilted down) in every test, and the ranging modules were used to measure distances. A total of 25 image sets and ranging data were collected, and the spatial coordinates of the laser light spots were simultaneously measured using the total station (Figure 8b).
(3) As the camera was in different locations and at different attitudes when capturing different images, laser light spots could not be used to calculate the laser beam vectors. Thus, the control point coordinates of the photos and collinear spatial resections were used to calculate the outer orientation parameters $\left(X_{L},Y_{L},Z_{L},\omega ,\phi ,\kappa \right)$ of the images. The outer orientation was used as a basis to translate and rotate the coordinate system of each photo to a coordinate system in the same space. The converted laser light spots were subsequently used to calculate the laser beam vectors and laser launch point coordinates. The collinearity equations are as follows:
  (1) $$x_{a}=x_{0}-f\left[\frac{m_{11}\left(X_{A}-X_{L}\right)+m_{12}\left(Y_{A}-Y_{L}\right)+m_{13}\left(Z_{A}-Z_{L}\right)}{m_{31}\left(X_{A}-X_{L}\right)+m_{32}\left(Y_{A}-Y_{L}\right)+m_{33}\left(Z_{A}-Z_{L}\right)}\right]$$
  (2) $$y_{a}=y_{0}-f\left[\frac{m_{21}\left(X_{A}-X_{L}\right)+m_{22}\left(Y_{A}-Y_{L}\right)+m_{23}\left(Z_{A}-Z_{L}\right)}{m_{31}\left(X_{A}-X_{L}\right)+m_{32}\left(Y_{A}-Y_{L}\right)+m_{33}\left(Z_{A}-Z_{L}\right)}\right]$$
  where f is the focal length of the camera, $x_{0}\;\mathrm{and}\;y_{0}\;\mathrm{are}$ coordinates of the principle point of a photo, $x_{a}\;\mathrm{and}\;y_{a}$ are photo coordinates of the corrected control point, $X_{L}\;\mathrm{and}\;Y_{L}\;\mathrm{and}\;Z_{L}$ are object spatial coordinates the camera projection center, $X_{A}\;\mathrm{and}\;Y_{A}\;\mathrm{and}\;Z_{A}$ are object spatial coordinates of the control point, $m_{ij}$ are rotation matrices composed of the image spatial rotation angles.

Two collinearity equations were derived for each control point using (1) and (2), where $X_{A},\;Y_{A},\;\mathrm{and}\;Z_{A}$ were known variables. Thus, six collinearity equations were derived from three control points and used to solve six unknown variables $\left(X_{L},Y_{L},Z_{L},\omega ,\phi ,\;\kappa \right)$, which were the focal points and attitudes of the camera when capturing photos. When the number of control points exceeded three, the least squares method was used to solve the six equations. As collinearity equations are not linear, they must be made linear using the Taylor series and solved using the iteration method.

**Figure 9:**
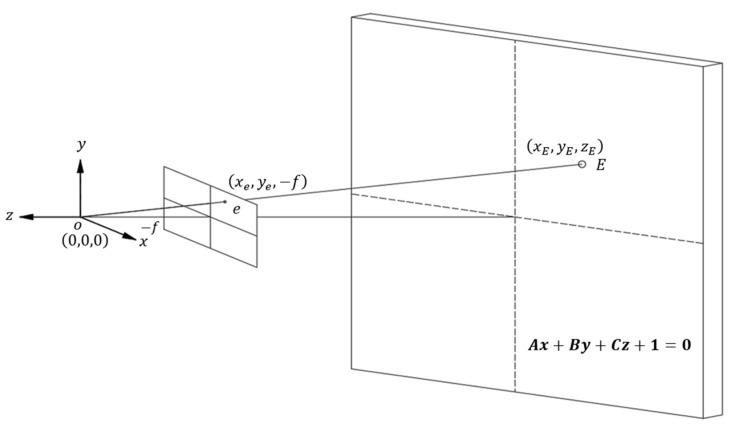
Spatial relationships between the photo projection vector and projection plane.

After calibrating the camera and laser ranging modules, photo coordinate systems were constructed with the focal points as origins. The laser ranging modules provided the spatial plane equations of the captured target surfaces, which could be used to calculate the spatial coordinates of object points (*E*) in subsequent measurements when combined with the plane equations and projection vectors of image points (*e*) (Figure 9).

Laser range finders could be used to calculate spatial projection plane equations, such as Equation (3).

(3) Ax+By+Cz+1=0 

The relationship between the focal point, image point, and object point can be described as follows:

(4) $$\vec{oe}=\left(x_{e},y_{e},-f\right)=\frac{\vec{oE}}{n}=\frac{\left(x_{E},y_{E},z_{E}\right)}{n}\;\;$$

where *o* is the focal point, *e* is the image point, *E* is the object point, $\vec{oe}$ is the vector from the focal point to the image plane, ($x_{e},y_{e},-f$) are the photo coordinates, *f* is the focal length, $\vec{oE}$ is the spatial vector from the focal point to the object point, $\left(x_{E},y_{E},z_{E}\right)$ are the object point coordinates, and n is the ratio of $\vec{oe}$ to $\vec{oE}$. By substituting (4) into (3), n can be calculated.

The object point’s spatial coordinates can be expressed as follows:

(5) $$E=o+\vec{oE}=\left(0,0,0\right)+\left(x_{E},y_{E},z_{E}\right)=n\left(x_{e},y_{e},-f\right)$$

By substituting the object point’s spatial coordinates into the object plane’s spatial equation, the following equation can be calculated:

(6) $$A\left(n\times x_{e}\right)+B\left(n\times y_{e}\right)+C\left(n\times -f\right)+1=0\;$$

Moreover, $n=\frac{-1}{A\times x_{e}+B\times y_{e}+C\times -f}$ can be used to calculate the object point’s spatial coordinates ($x_{E},y_{E},z_{E}$).

#### 2.2.4. Indoor Measurement Accuracy Tests for the Camera and Laser Ranging Modules

**Figure 10:**
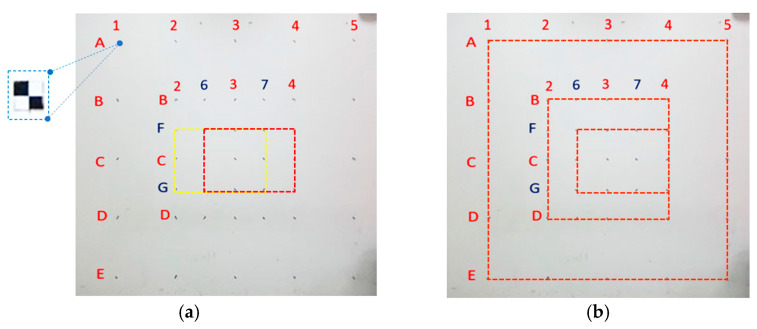
Control point distribution. (**a**) The same dimensions of the rectangular box (**b**) Different dimensions of the rectangular box.

Control points were marked on an indoor wall (Figure 10) to determine the measurement accuracy of the camera and laser ranging modules after calibrations. The horizontal from 1 to 7 and vertical from A to G mark the location of the control points. The location description rule is A1, A2, A3, A4, A5, and the other five points in the horizontal direction of A. The horizontal direction of B has seven points: B1, B2, B6, B3, B7, B4, B5, etc. There are 41 control points in total. The coordinates of the 41 points are measured by total station (measurement accuracy of ±1mm; this also means that the credibility of the value in Table 3 and Table 4 is 1 mm) and used as the true value when analyzing the accuracy of the image projection measurements of this system. The results of the related experimental test and data analysis are as follows.

By maintaining constant dimensions of the four sides of a rectangular box (i.e., 0.50 m × 0.75 m, Figure 10a) and increasing the photo-shooting distance from 1 to 3 m in 1-m increments, this study found that the higher the photo-shooting distance, the higher the measurement error (error is projection measurement value minus true value). At a distance of 3 m, the maximum and relative errors were 6 mm and 0.8%, respectively (Table 3).

By maintaining a constant distance (i.e., 3 m) and increasing the dimensions of the rectangular box (i.e., from 0.50 m × 0.75 m to 1.0 m × 1.0 m and 2.0 m × 2.0 m, Figure 10b), this study found that the larger the rectangular box, the higher the measurement errors. With dimensions of 1.0 m × 1.0 m, the maximum and relative errors were 7 mm and 0.7%, respectively. Moreover, with dimensions of 2.0 m × 2.0 m, the maximum and relative errors were 19 mm and 1.0%, respectively (Table 4).

Summarizing the experimental results using the developed detection camera, the indoor accuracy tests conducted in this study revealed that when the dimensions of the rectangular box were kept constant, the inspection camera exhibited higher measurement errors as the measurement distance increased. At a measurement distance of 3 m, the error and relative error were <6 mm and 0.8%, respectively. By contrast, when the measurement distance was kept constant at 3 m, the inspection camera exhibited a higher measurement error as the dimensions of the rectangular box increased. For example, when the dimensions of the rectangular box were 1.0 m × 1.0 m, the error and relative error were <7 mm and 0.7%, respectively. When the dimensions of the rectangular box increased to 2.0 m × 2.0 m, the error and relative error were 19 mm and 9%, respectively. Thus, when using the developed UAV to perform bridge inspections, users should ensure that the measurement distance does not exceed 3 m and that the target range does not exceed 1.0 m × 1.0 m. During tests in the lab environment, we used homemade control points and pasted them on the wall; they were designed to be 20 mm by 20 mm and checkered with black and white, so that we could explicitly recognize the central position of the control point. Additionally, we aimed to improve the accuracy of measuring the center position of the control point. To explore the measurement accuracy of the camera and laser ranging module, we separately executed far and close distances and different measurement ranges in the image to find their influence on the measuring error, so that we could understand the best working distance. However, the fly in the ointment is the fact that we did not execute the visual test using a UAV. Thus, we recommended that follow-up researchers create some homemade crack images on the wall, so they can explore the pros and cons of the visual and UAV means and provide further research.

### 2.3. Crack Identification and Measurement Methods

**Figure 11:**
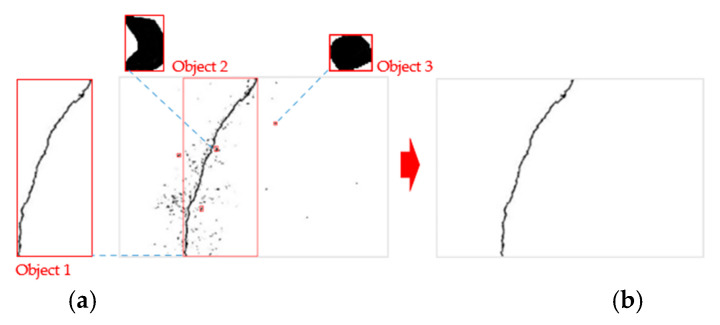
Illustration of noise removal. (**a**) Object’s rectangle frame (**b**) Result.

**Figure 12:**
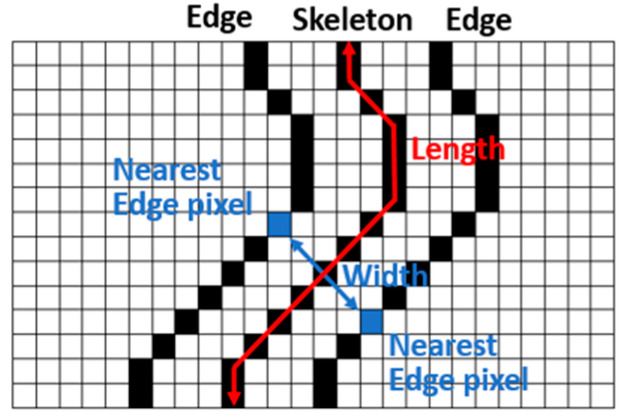
Illustration of crack width and length calculations [23].

In order to effectively extract the surface cracks in concrete bridge components, the Sauvola local thresholding method and two optimal parameter sets were used to produce binary images of cracks according to the recommendations of Kim et al. [21]. After the binary images were produced, due to stains or potholes in the concrete surface, in addition to the linear objects (cracks), there were also dot-like and plane-like objects in the image (the stains or potholes are regarded as noise). A rectangular frame was drawn for each object in the image according to the maximum range of the shape and the length–width ratio of the rectangle and density of the object in the rectangle were calculated. The “trial-and-error method” was used to determine the threshold of the two to remove the dot-like and plane-like noise in the image (Figure 11). The main crack skeleton and crack outline were calculated with the Zhang–Suen skeletonization algorithm and the erosion algorithm in morphology. Then, we could confirm the crack location in the image (Figure 12).

In this study, we used an 8-bit color image for testing and calculations, and we directly read the value of the pixel (0–255) in the image, and all the image pixels (x, y) that contained the data of matrices R(x, y), G(x, y), and B(x, y). Although the color images could easily be identified by the human eye, they could not be identified by machines because the various matrices increased the image processing time and image complexity. Therefore, prior to image processing, RGB images were converted into grayscale [I(x, y)] images to facilitate image processing. The weighted grayscaling formula of Dorafshan [10] was used to produce grayscaling results similar to the sensitivity of the human eye.

(7) I(x,y)=0.2989R(x,y)+0.5870G(x,y)+0.1140B(x,y)

Image thresholding entails dividing the grayscale intensity of image pixels according to selected threshold values. All the pixels in an image are divided into foreground pixels (necessary pixels) and background pixels (unnecessary pixels). The quality of this thresholding process depends on the selected threshold values. Thresholding algorithms can be classified as single-thresholding algorithms and global thresholding algorithms (or fixed thresholding algorithms). The most representative global thresholding algorithm is Otsu’s method. Local thresholding algorithms (or dynamic thresholding algorithms), such as the Sauvola local thresholding algorithm, change when changes occur in local pixels. In general, local thresholding algorithms outperform global thresholding algorithms when an image has uneven illumination. In crack image processing, the Sauvola local thresholding method outperforms Otsu’s method [28]. The equation for the Sauvola local thresholding method is as follows [29]:

(8) $$T\left(x,y\right)=m\left(x,y\right)\left[1+k\left(\frac{s\left(x,y\right)}{R}-1\right)\right]\;$$

where T(x,y) is the threshold value obtained through calculation, m(x,y) is the mean grayscale intensity of a pixel in the thresholding window whose dimensions are set by the user, s(x,y) is the standard deviation in the grayscale intensity inside the window, *R* is the dynamic range for normalizing s(x,y), *k* is the sensitivity when controlling the contribution of the statistical parameters.

The threshold value T(x,y) is determined by users, using the window sizes and sensitivities set. Thus, selecting appropriate window sizes and sensitivities can produce accurate thresholding results. Kim et al. used a set of optimal detection parameters that included the minimum crack length and crack width identification errors. The optimal detection parameters for crack length were a window size of 180 by 180 pixels and a sensitivity (*k*) of 0.18, whereas those for crack width were a window size of 70 by 70 pixels and a sensitivity of 0.42. Accordingly, these two parameter sets were used in this study to produce binary images of the crack length and width.

#### 2.3.1. Extracting the Length of the Main Crack Skeleton

In this study, binary crack images were skeletonized, and unnecessary skeleton branches were removed to extract the main cracks and calculate crack lengths. The skeletonization of cracks enables images to be refined, the central axes in images to be accentuated, and image directions to be retained. In the present study, the Zhang–Suen algorithm was adopted for skeletonization [30]. This algorithm is a sub-iteration algorithm comprising two steps. Each iteration continues until all pixels are filtered out. When an odd number of iterations is performed, pixels in the right, bottom, and upper left corners of images are removed. When an even number of iterations is performed, pixels in the left, top, and bottom right corners of images are removed. The pixel removal conditions are identical for odd and even iteration numbers.

**Figure 13:**
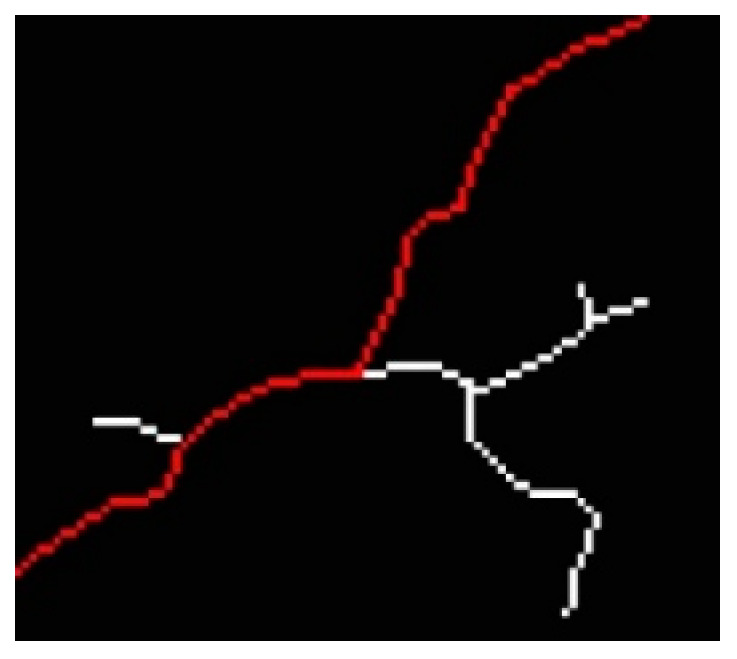
Main crack skeleton.

After skeletonizing the cracks, the skeletons comprise nodes and line segments, and the structures of the skeletons resemble those of tree diagrams. However, because cracks are small gaps, the skeletons form closed loops that tree diagrams do not contain. The main crack axes are generally the paths connecting the two end points that are the farthest apart. Such paths have the same definition as the diameters in tree structures, in which diameters are the paths between the two end points that are farthest from each other [31]. The diameters in such structures are determined by first finding the shortest paths between end points and then identifying the longest paths. In the present study, the aforementioned method was adopted to extract the main crack axes and use them to calculate crack lengths. Crack “skeletons” were disassembled into line segments from connection points to connection points or from connection points to end points, and the length of each line segment was recorded. Dijkstra’s shortest path algorithm [32] was then used to calculate the shortest paths among all end points to select the main crack axes. This algorithm searches for the shortest path between two points, beginning from the starting point to the perimeter vertices and finally reaching the end points. The paths between different points can be assigned different weights to indicate their different lengths. Finally, the longest path among all the main crack skeleton paths was selected as the main crack skeleton (Figure 13), and the length of this skeleton was calculated. A conversion was conducted using aspect ratios to calculate the actual crack length.

#### 2.3.2. Extraction of Crack Widths

Edge detection algorithms can be used to extract crack outlines. These algorithms extract crack outlines using the erosion method, which is explained in the following text. First, the foreground pixels of an image are eroded using appropriate structural elements. Next, the eroded pixels are subtracted from the pre-erosion foreground pixels to calculate the crack outline. After extracting the crack outline, the user searches for the outline pixel whose two sides are closest to a pixel of the main crack skeleton to measure the width of the crack at this pixel. Subsequently, a conversion is performed according to the spatial resolution of the image to calculate the actual crack width. This conversion is expressed as follows:

(9) $$w=w_{p}\times \mathrm{SR}$$

where w is the real crack width in metric units (mm), $w_{p}$ is the calculated crack width in pixels, and SR is the image’s spatial resolution.

## 3. Crack Inspection Efficiency and Accuracy of the Developed UAV System

### 3.1. Outdoor Bridge Inspection Tests

**Figure 14:**
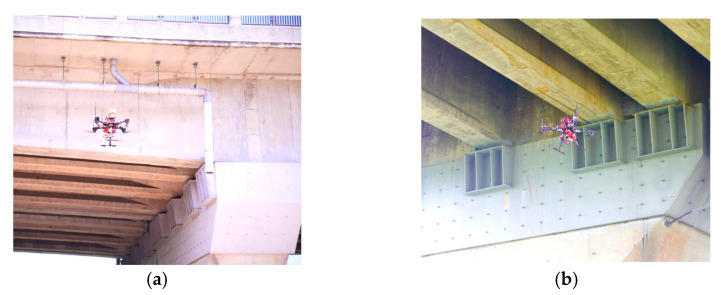
Bridge inspections performed by the self-assembled UAV: (**a**) operation on the side of the Wuling Bridge; (**b**) operation at the bottom of the Wuling Bridge.

Outdoor bridge inspections were performed at Wuling Bridge, which is a highway bridge that runs across a river in Taoyuan, Taiwan. The developed UAV, which contains a camera and laser ranging modules, has a double-lens design. The control lens of the UAV is a fisheye lens that creates wide views to control the flight of the UAV. By contrast, the ranging camera, which was developed in this study and can be controlled by a user, is used to perform bridge structure inspections. To monitor the UAV flight situations in real time, a wireless numerical transmission system is used to send flight information to a ground monitoring station. The flight information is monitored at all times to ensure flight safety. The self-assembled UAV is equipped with a photo-shooting and ranging system to capture photos of bridge cracks and damages. The bridge inspection camera installed on the tripod head can rotate vertically and horizontally, enabling the UAV to complete all inspections of the sides and bottom of bridges in a single attempt, which results in a high bridge inspection efficiency. The outdoor bridge inspection operations of the developed UAV are illustrated in Figure 14a,b.

### 3.2. Outdoor Bridge Inspection Tests

(1) Case 1

**Figure 15:**
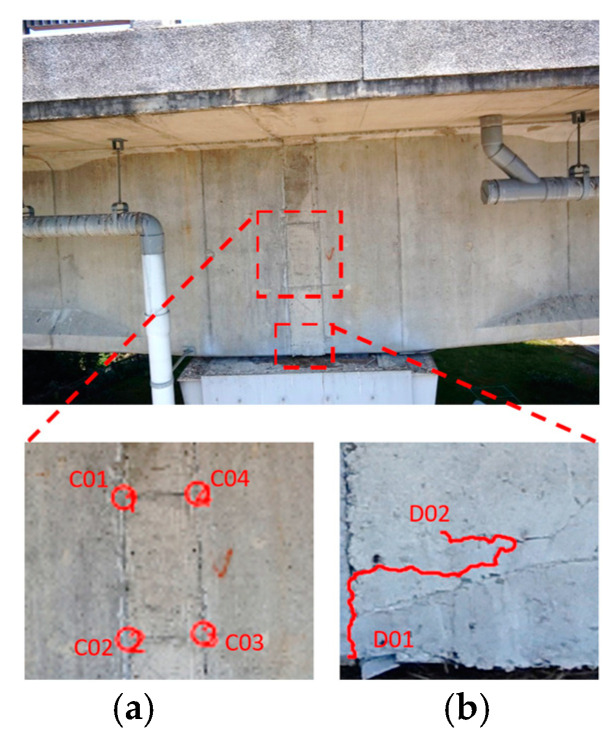
Damage on the side of the main beam at the test site: (**a**) connected concrete cracks; (**b**) unconnected concrete cracks.

**Figure 16:**
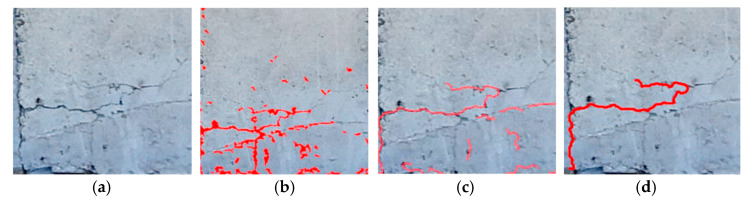
Crack identification: (**a**) original image; (**b**) binary image; (**c**) image obtained after noise removal and skeletonization; (**d**) main cracks.

According to the captured images of the Wuling Bridge (file name: DSC00123), it contained two areas of severe damage on the side of the main beam (Figure 15). The photo-shooting times for the top left, bottom left, bottom right, and top right rangefinder data were 4.694, 4.659, 4.637, and 4.676 m, respectively. The captured images were used to detect cracks, after which aspect ratios were used to convert the data into crack lengths and widths. As cracks have lower grayscale values than their backgrounds and are distorted, shape and grayscale value were used to set crack identification rules and remove non-crack regions. The crack identification results are shown in Figure 16.

Ranging data were used to project the calculated images onto actual object planes to enable the measurement of crack dimensions. Table 5 presents the measured data. The connected concrete cracks had a length and width of approximately 0.738 m and 0.355 m, respectively, whereas the unconnected concrete cracks had a length and width of approximately 0.412 m and 3.9 mm, respectively. To analyze the measurement accuracy, comparisons were conducted between the left- and right-side lengths and between the bottom- and top-side lengths of the connected concrete cracks. The corresponding errors were 11 and −23 mm, respectively, and the corresponding relative errors were 1.5% and 6.1%, respectively. The errors increased considerably because the photo-shooting distance was 4.6 m (Table 6).

(2) Case 2

**Figure 17:**
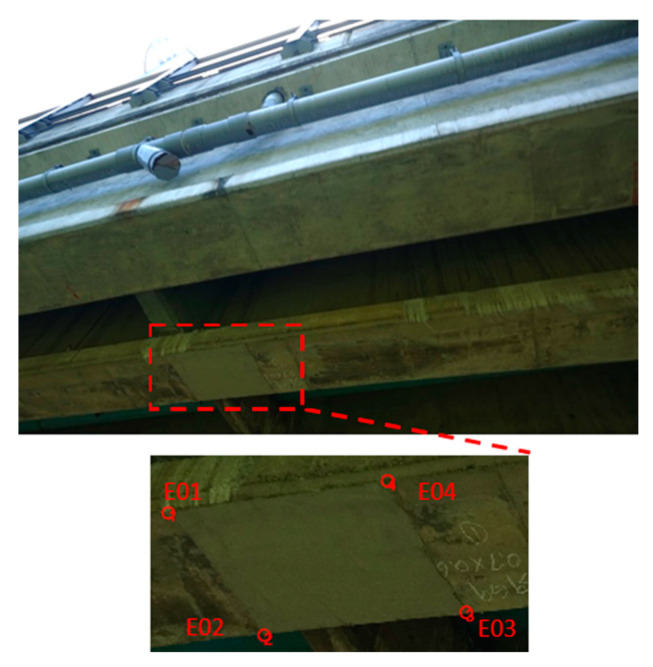
Damage at the bottom of the main beam at the test site: Refilled concrete.

According to the images captured of the Wuling Bridge, the bridge (file name: DSC00358) contained two areas of severe damage at the bottom of the main beam (Figure 17). The photo-shooting times for the top left, bottom left, bottom right, and top right rangefinder data were 2.930, 2.969, 2.927, and 2.888 m, respectively.

Ranging data were used to project the images onto actual target planes to allow the measurement of the dimensions of the cracks. Table 7 presents the measured data that were calculated for Case 2. The part of the bridge with refilled concrete had a length and width of 0.638 m × 0.910 m. To analyze measurement accuracy, comparisons were conducted between the left- and right-side lengths and between the bottom- and top-side lengths of the refilled concrete. The corresponding errors were 19 and 17 mm, respectively, and the corresponding relative errors were 3.0% and 1.8%, respectively. The errors became smaller because the photo-shooting distance was shortened to 2.9 m (Table 8).

(3) Case 3

**Figure 18:**
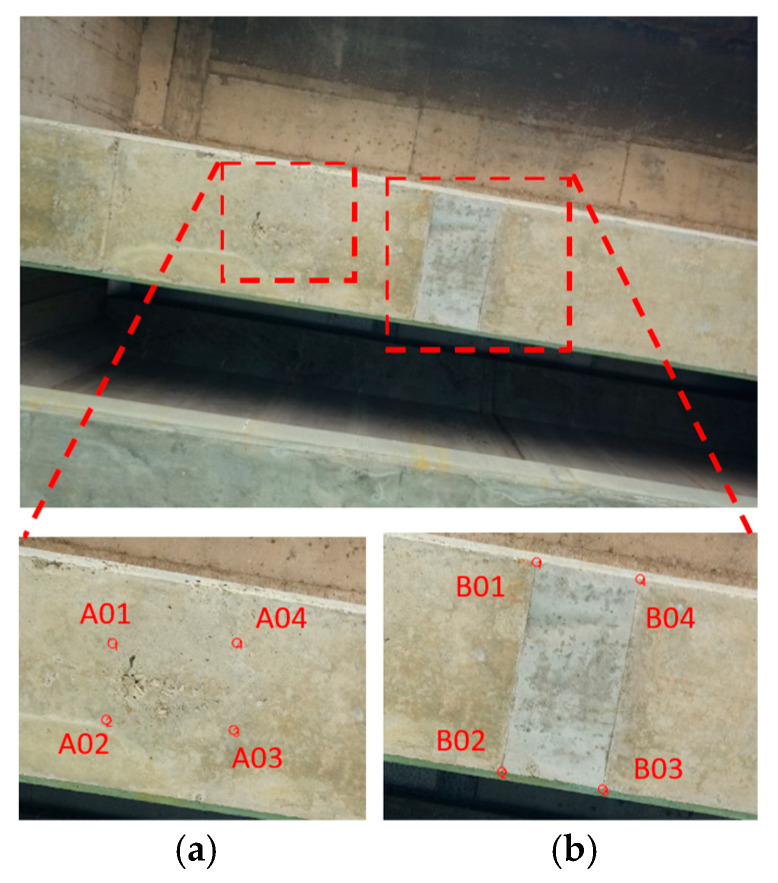
Damage at the bottom of the main beam at the test site: (**a**) incomplete concrete grouting (hive phenomenon); (**b**) refilled concrete.

According to the images of the Wuling Bridge, the bridge (file name: DSC00451) contained two areas of severe damage at the bottom of the main beam (Figure 18). The photo-shooting times for the top left, bottom left, bottom right, and top right rangefinder data were 2.506, 2.515, 2.515, and 2.508 m, respectively.

Ranging data were used to project the images onto actual target planes to allow for measurement of the crack dimensions. Table 9 presents the measured data that were calculated for Case 2. The part of the bridge with an incomplete concrete grouting had a length and width of approximately 0.23 and 0.383 m, respectively, whereas the part of the bridge with refilled concrete had a length and width of 0.638 m × 0.319 m, respectively. To analyze measurement accuracy, comparisons were conducted between the left- and right-side lengths and between the bottom- and top-side lengths of the refilled concrete. The corresponding errors were −5 and 0 mm, respectively, and the corresponding relative errors were 0.8% and 0.0%, respectively. The errors were less than 5 mm because the photo-shooting distance was 2.5 m (Table 10).

Summarizing the experimental results, Case 1 of the outdoor bridge inspection tests indicated that the developed UAV can automatically conduct crack identification and crack size measurements. Although a photo-shooting distance of 4.6 m produced a relatively large measurement error of 6.1%, the UAV could still identify cracks and calculate the crack length (0.412 m) and width (3.9 mm; 1.3 mm/pixel). Case 2 of the outdoor inspection tests revealed the refilled concrete at the bottom of the main beam. The UAV could directly mark the range of this grouting and calculate its size. The errors became smaller because the photo-shooting distance was shortened to 2.9 m. Case 3 of the outdoor inspection tests revealed the existence of incomplete concrete grouting at the bottom of the main beam. The UAV could directly mark the range of this grouting and calculate its size. At a photo-shooting distance of 2.5 m, the relative error was <0.8%.

## 4. Conclusions

In this study, a UAV was developed for omnidirectional bridge inspection. This UAV can be used to inspect all types of bridges. It can reach bridge sides and low bridge areas, stay near bridge components, and capture complete and clear images of all bridge parts. The camera of the developed UAV has a dual-lens design, where one lens is used for bridge inspections and another lens is used for flight control. The flight control lens was fixed to the body of the UAV to inform users about the dynamic conditions of the aircraft, thereby ensuring bridge inspection safety. By contrast, the inspection lens is operated by inspection personnel to inspect bridge cracks. As the camera is placed on a tripod head that can rotate horizontally and vertically, the developed UAV can complete inspections for all side and bottom surfaces of a bridge in a single attempt, which helps overcome the problems involved in traditional bridge inspection techniques and increases the bridge inspection efficiency.

We designed and developed an inspection camera that integrates the camera and the laser ranging module. Then, four sets of laser ranging modules were installed next to the camera to measure the working distance from the camera to the object and the projection plane equation of the object’s surface was calculated. Moreover, we accurately calculated the position of the UAV inspection camera relative to the bridge and overcame the limitations of vertical photography. Finally, great results were obtained in terms of crack recognition and measurements during the indoor and outdoor verification tests.

The aforementioned results indicate that the developed UAV system, which includes laser ranging modules, can effectively be used to conduct accurate bridge crack inspections. The results verified the measurement accuracy and inspection efficiency of the UAV. In the future, the effectiveness and accuracy of the UAV-based bridge inspection method adopted in this study can be improved with advances in relevant equipment, such as UAVs, cameras, ranging modules, and embedded systems.

## Figures and Tables

**Table 1:** Specifications of original UAV and inspection cameras.

Items	Original UAV Camera	Inspection Camera
Resolution (pixels)	976 × 494	4800 × 3200
Focus length (mm)	2.5	9.346
Image sensor size (inch)	1/3	1
Pixel size (mm)	0.007743	0.00275

**Table 2:** Camera elements of interior orientation and lens distortion calculation results.

Item	Parameter Name	Parameter	Value
Elements of interior orientation (mm)	Focal length	*f*	9.346
	Principal point	*x_o_*	6.442
		*y_o_*	4.506
Lens distortion	Radial distortion coefficients	*k*1	0.0120
		*k*2	−0.0229
	Tangential distortion coefficients	*p*1	0.0044
		*p*2	−0.0021

**Table 3:** Range size and error analyses at measurement distances of 1, 2, and 3 m.

Photo-Shooting Distance	Side Length Lo-Cation	True Value (m)	Projection Measurement Value (m)	Error (m)	Relative Error
1.0 m	F2~G2	0.500	0.499	0.001	0.2%
	G2~G7	0.741	0.737	0.004	0.5%
	G7~F7	0.500	0.499	0.001	0.2%
	F7~F2	0.739	0.738	0.001	0.1%
2.0 m	F6~G6	0.500	0.503	0.003	0.6%
	G6~G4	0.760	0.760	0.000	0.0%
	G4~F4	0.500	0.502	0.002	0.4%
	F4~F6	0.759	0.760	0.001	0.1%
3.0 m	F6~G6	0.500	0.499	0.001	0.2%
	G6~G4	0.760	0.758	0.002	0.3%
	G4~F4	0.500	0.499	0.001	0.2%
	F4~F6	0.759	0.753	0.006	0.8%

**Table 4:** Error analyses for different dimensions of the rectangular box.

Dimensions of the Rectangular Box	Side Length Location	True Value (m)	Projection Measurement (m)	Error (m)	Relative Error
0.50 m × 0.75 m	F6~G6	0.500	0.499	0.001	0.2%
	G6~G4	0.760	0.758	0.002	0.3%
	G4~F4	0.500	0.499	0.001	0.2%
	F4~F6	0.759	0.753	0.006	0.8%
1.0 m × 1.0 m	B2~D2	0.999	0.996	0.003	0.3%
	D2~D4	1.003	1.000	0.003	0.3%
	D4~B4	1.001	0.998	0.003	0.3%
	B4~B2	0.997	0.990	0.007	0.7%
2.0 m × 2.0 m	A1~E1	2.001	1.996	0.005	0.2%
	E1~E5	1.997	2.001	0.004	0.2%
	E5~A5	2.004	1.996	0.007	0.4%
	A5~A1	1.999	1.980	0.019	1.0%

**Table 5:** Analyses of the damage at the bottom of the main beam.

**Rectangular Box Location**	**Coordinates (*x*, *y*, *z*) (m)**	**Side Location**	**Length (m)**	**Damage Situation**
C01	(−0.141, −0.264, −4.700)	Left	0.738	Connected concrete crack
C02	(−0.108, −0.912, −4.348)	Bottom	0.355	
C03	(0.245, −0.880, −4.324)	Right	0.727	
C04	(0.236, −0.240, −4.669)	Top	0.378	
**Location**	**Coordinates (*x, y, z*) (m)**	**Crack Length (m)**	**Crack Width (mm)**	**Damage Situation**
Starting point (D01)	(0.477, −0.996, −4.233)	0.421	3.9	Unconnected concrete cracks
End point (D02)	(0.615, −0.904, −4.266)			

**Table 6:** Accuracy analysis for side length measurement at a photo-shooting distance of approximately 4.6 m.

Side Location	Length (m)	Error (m)	Relative Error	Damage Situation
Left	0.738	0.011	1.5%	Connected concrete crack
Right	0.727			
Bottom	0.355	0.023	6.1%	
Top	0.378			

**Table 7:** Damage analysis at the bottom of the main beam.

Location	Coordinates (*x, y, z*) (m)	Side Location	Length (m)	Damage Situation
E01	(−1.408, −1.217, −4.163)	Left	0.638	Refilled concrete
E02	(−1.140, −1.744, −4.403)	Bottom	0.910	
E03	(−0.338, −1.528, −4.031)	Right	0.619	
E04	(−0.584, −1.013, −3.791)	Top	0.927	

**Table 8:** Accuracy analysis for side length measurement at a photo-shooting distance of approximately 2.9 m.

Side Length Location	Length (m)	Error (m)	Relative Error	Damage Situation
Left	0.638	0.019	3.0%	Refilled concrete
Right	0.619			
Bottom	0.910	0.017	1.8%	
Top	0.927			

**Table 9:** Damage analysis at the bottom of the main beam.

Location	Coordinates (*x, y, z*) (m)	Side Location	Length (m)	Damage Situation
A01	(−0.601, 0.211, −2.487)	Left	0.230	Incomplete concrete grouting (hive phenomenon)
A02	(−0.627, −0.016, −2.514)	Bottom	0.383	
A03	(−0.245, −0.049, −2.523)	Right	0.264	
A04	(−0.232, 0.213, −2.492)	Top	0.369	
B01	(0.303, 0.271, −2.491)	Left	0.638	Refilled concrete
B02	(0.204, −0.355, −2.566)	Bottom	0.319	
B03	(0.518, −0.408, −2.576)	Right	0.643	
B04	(0.618, 0.223, −2.501)	Top	0.319	

**Table 10:** Accuracy analysis for side length measurement at a photo-shooting distance of approximately 2.5 m.

Side Length Location	Length (m)	Error (m)	Relative Error	Damage Situation
Left	0.638	0.005	0.8%	Refilled concrete
Right	0.643			
Bottom	0.319	0.000	0.0%	
Top	0.319			

## Data Availability

Not applicable.
